# *Parkia platycephala* pod meal as a total replacement for Tifton-85 hay in high-concentrate diets for lambs: ingestive behavior, physiological, and metabolic parameters

**DOI:** 10.1007/s11250-026-04850-z

**Published:** 2026-01-30

**Authors:** Henrique N. Parente, Vanilsa C. Sousa, Gabrielle M. Oliveira, Osmar M. F. Neto, Gisele T. A. Silveira, Aylpy R. D. Santos, Juliana D. Oliveira, Anderson M. Zanine, Daniele J. Ferreira, Miguel A. Moreira Filho, Michelle O. M. Parente

**Affiliations:** 1 Federal University of Maranhão, Chapadinha, MA Brazil; 2 Mato Grosso do Sul State University, Aquidauana, MS Brazil; 3 Federal University of Grande Dourados, Dourados, MS Brazil; 4 Federal University of Piauí, Teresina, PI Brazil

**Keywords:** Non-fibrous carbohydrates, Energy, Faveira, Fiber, Rumination time, Urinalysis

## Abstract

This study aimed to evaluate the effects of the total replacement of Tifton-85 hay with faveira (*Parkia platycephala*) pod meal on the ingestive behavior, physiological, and metabolic parameters of finishing lambs fed high-concentrate diets (faveira pod meal, 30%; whole corn grain, 20%; soybean meal, 14.5%; wheat bran, 33.2%; mineral salt, 2% and limestone, 0.3%). Fourteen Santa Inês lambs, 150 days old and with an initial body weight of 21 ± 2.4 kg, were assigned in a completely randomized design to two treatments (DTH: diet with Tifton-85 hay and DFP: diet with faveira pod meal replacing Tifton-85 hay), with seven replicates. Lambs fed the DTH diet spent more time ruminating (*P* < 0.001). Physiological parameters of the lambs did not differ according to the experimental diets (*P* ≥ 0.209). It was observed that the DFP diet reduced water intake (*P* = 0.048), water intake from feed (*P* = 0.029), and total water intake (*P* = 0.045). DFP diet resulted in higher plasma glucose concentration (*P* = 0.005), whereas the DTH led to an increase in urea concentration (*P* = 0.023). The DFP diet also resulted in a lower urine pH (*P* = 0.004). Faveira pod meal can totally replace Tifton-85 hay in high-concentrate diets for finishing lambs, as it does not compromise ingestive behavior or physiological parameters and leads to better nutrient utilization from the diet.

**Supplementary Information:**

The online version contains supplementary material available at https://doi.org/10.1007/s11250-026-04850-z.

## Introduction

Among the factors limiting sheep meat production in semiarid regions, forage seasonality is one of the most significant (Silva et al. 2022; Souza et al. 2024). The low availability of feed during the dry season limits animal performance, leading to a decrease in the continuous supply of meat from this specie. In this context, feedlot represents an important strategy, allowing producers to finish animals in a continuous and faster manner, obtaining better-finished carcasses and superior meat quality (Paim et al. 2011).

In feedlot diets, a high proportion of concentrate is commonly used, which considerably increases feeding costs and, in some cases, can represent up to 70% of the total costs of this system (Lima et al. 2017). To ensure the maintenance of ruminal health, it is necessary to use good-quality roughage as a source of physically effective fiber. However, some roughage sources, such as Tifton-85 hay, which is widely used in lamb feedlots, should have their use limited due to their higher energy cost (Suzuki et al. 2014; Weiss et al. 2017). Furthermore, poor pasture management often leads this ingredient to have low protein content and a high concentration of low-quality fiber, limiting the nutritional efficiency of the feedlot system.

Therefore, it becomes relevant to investigate alternative sources to roughages in diets for feedlot sheep, aiming to increase profitability. In this context, faveira pod meal (*Parkia platycephala*) stands out as an important ingredient for supplementing animals during periods of pasture scarcity (Silva et al. 2019). Recent studies have shown that this alternative feed can replace corn in ruminant diets (Araújo et al. 2019; Batista et al. 2020; Gomes et al. 2024; Lima et al. 2024, 2025). However, there are no reports in the literature of studies that have validated the use of faveira pod meal as a substitute for roughage in sheep diets. Our hypothesis is that faveira pod meal, when combined with whole corn grain as a source of physically effective fiber, eliminates the need for roughage in feedlot diets for sheep. In this context, the evaluation of ingestive behavior, together with the analysis of physiological and metabolic parameters, can provide essential insights for validating this feeding strategy.

Thus, this study aimed to evaluate the total replacement of Tifton-85 hay with faveira pod meal in high-concentrate diets for finishing lambs, assessing ingestive behavior, physiological, and metabolic parameters.

## Materials and methods

### Location and procedures

The experiment was conducted at the Small Ruminant Sector of the Center for Agricultural Sciences (CCCh) of the Federal University of Maranhão (UFMA), located in the municipality of Chapadinha (3° 44′ 31″ S, 43° 21′ 36″ W, at an altitude of 93 m). According to the Köppen classification, the climate of Chapadinha is classified as tropical hot and humid (Aw), with annual average temperatures exceeding 27 °C.

The experiment was approved by the Ethics Committee on the Use of Animals of UFMA under protocol number 23115.005618/2023-79.

### Animals, management, experimental diets and intake evaluation

A total of fourteen castrated male Santa Inês lambs, with an initial average body weight (IBW) of 21 ± 2.4 kg and approximately 150 days of age, were assigned to a completely randomized design with two treatments and seven replicates. The IBW of the animals was used as a covariate. The experimental period lasted 60 days, with the first 10 days allocated for animal adaptation to the facilities, diets, and management, and the remaining 50 days for data collection.

At the beginning of the experimental period, the animals were weighed, identified, dewormed (2 mL of Closantel), and individually housed in metal pens of 1.45 m^2^, with concrete floors and equipped with a feeder and a drinker.

The chemical composition of the ingredients used for diet formulation is presented in supplementary material. The treatments consisted of two diets (Table 1): DTH (diet with Tifton-85 hay) and DFP (diet with faveira pod meal replacing Tifton-85 hay). The diets were formulated based on the nutritional requirements prescribed by the National Research Council (NRC 2007) for lambs with an expected weight gain of 200 g/day, considering a dry matter intake (DMI) of 3.5% of body weight (BW). The average particle size of the diets was determined using a *Penn State* particle separator (supplementary material).

**Table 1:** Proportion of ingredients and chemical composition of the experimental diets

Ingredients (%)	Diets	
	DTH	DFP
Tifton-85 hay	30.00	0.00
Faveira pod meal	0.00	30.00
Whole corn grain	0.00	20.00
Corn ground	20.00	0.00
Soybean meal	16.70	14.50
Wheat bran	31.00	33.20
Mineral salt	2.00	2.00
Limestone	0.30	0.30
Chemical composition (%)		
Dry matter	85.28	85.78
Ash	5.53	4.12
Organic matter	94.47	95.88
Neutral detergent fiber	37.88	19.99
Crude protein	16.14	15.78
Total carbohydrates	73.29	74.54
Non-fibrous carbohydrates	35.41	54.55
Ether extract	5.04	5.56
Metabolizable energy (Mcal/kg)	2.81	3.03

DTH, diet with Tifton-85 hay; DFP, diet with faveira pod meal replacing Tifton-85 hay

Composition of the mineral supplement per kilogram of product: Sodium, 147 g; Calcium, 120 g; Phosphorus, 87 g; Sulfur, 18 g; Zinc, 3,800 mg; Iron, 1,800 mg; Manganese, 1,300 mg; Fluoride, 870 mg; Copper, 590 mg; Molybdenum, 300 mg; Iodine, 80 mg; Cobalt, 40 mg; Chromium, 20 mg; Selenium, 15 mg

The animals were fed twice daily, at 08:00 and 16:00 h. The amount of feed offered was adjusted daily to allow for 10% orts, ensuring *ad libitum* intake for the lambs. For this purpose, before the morning feeding, the orts were manually collected from the feeders and weighed. Every three days, samples of the orts and the offered diets were collected, identified, and stored in a freezer at -18 °C for subsequent chemical analysis and determination of dry matter intake (DMI) and neutral detergent fiber intake (NDFI), calculated as the difference between the amount in the diet and in the orts.

At the end of the experimental period, samples of ingredients, diets, and orts were thawed and ground in a Wiley-type knife mill equipped with 1-mm sieve screens. After grinding, the contents of dry matter (DM; 934.01), crude protein (CP; 981.10), ether extract (EE; 920.39), and ash (934.05) were determined according to the methodologies proposed by Association of Official Analytical Chemists (AOAC 1990). The neutral detergent fiber (NDF) content was obtained according to the method proposed by Van Soest et al. (1991). Organic matter (OM) was calculated as the difference between total DM and ash, while total carbohydrates (TC) were calculated using the equation proposed by Sniffen et al. (1992): TC = 100 − (%CP + %EE + %ash).

The non-fibrous carbohydrates (NFC) were estimated using the formula proposed by Hall (2003): NFC = %TC − %NDF. The metabolizable energy (ME) concentration was estimated considering that 1 kg of total digestible nutrients (TDN) corresponds to 4.409 Mcal of digestible energy (DE), and 1 Mcal of DE is equivalent to 0.82 Mcal of ME (NRC 2007). It is important to note that the TDN content was obtained using the formula described by Weiss et al. (1992): TDN = DCP + DNDF + DNFC + (DEE × 2.25), where DCP, DNDF, DNFC, and DEE represent the digestible fractions of CP, NDF, NFC, and EE, respectively.

### Ingestive behavior

On the thirty-eighth day of the experimental period, the ingestive behavior of the animals was evaluated using the instantaneous *scan sampling* method proposed by Johnson and Combs (1991), at five-minute intervals for 24 continuous hours, starting at 08:00 h. The evaluations were conducted by two previously trained observers, who recorded, in ethograms, the following behaviors: feeding, defined as the animal going to the feeder, including feed grasping, chewing, and swallowing; rumination, defined as the activity corresponding to regurgitation, resalivation, rechewing, and reswallowing of the bolus; and idle, when the animals were not performing any specific activity. The time spent on these activities was expressed in hours and as a percentage.

The feeding and rumination efficiencies of DM and NDF were calculated according to the model proposed by Polli et al. (1996): FEDM = DMI/FT, where FEDM is the feeding efficiency in DM intake (g/h) and FT is the feeding time; FENDF = NDFI/FT, where FENDF is the feeding efficiency in NDF intake (g/h); RUMDM = DMI/RT, where RUMDM is the rumination efficiency of DM (g/h) and RT is the rumination time; RUMNDF = NDFI/RT, where RUMNDF is the rumination efficiency of NDF (g/h).

### Physiological parameters and water intake

For the evaluation of the animals’ physiological parameters, measurements of respiratory rate (RR), forehead temperature (FOT), flank temperature (FLT), tail temperature (TT), surface temperature (ST), and rectal temperature (RT) were taken. These parameters were recorded daily, between the thirty-first and thirty-sixth day of the experiment, at four different times: 6:00, 10:00, 14:00, and 18:00 h.

The RR values were obtained by counting the number of right flank movements of the animals over a 15-second period using a digital stopwatch, according to the methodology proposed by Kawabata et al. (2013). To determine the number of movements per minute, the recorded RR values were multiplied by four. FOT, FLT, and TT were measured using an infrared digital thermometer (Akrom KR380, Porto Alegre, RS, Brazil) positioned 15 cm away from the animals. Based on these measurements, ST was calculated as the average of these three temperatures. RT was determined using a digital clinical thermometer (Incoterm Termo Med 1.0, São Paulo, SP, Brazil), which was inserted into the rectum of each lamb and kept in place for two minutes until temperature stabilization (Santos et al. 2019).

Water intake (WI) was determined between the thirty-first and thirty-fifth day of the experimental period, calculated as the difference between the quantity supplied, the daily offer and the average daily evaporation. During this period, three buckets were placed in different locations within the feedlot barn, without access to the animals, to measure the amount of water lost through evaporation. The average amount of water lost from these buckets was considered the evaporation loss. The water intake from feed (WIF) was determined by the difference between the intake of fresh matter and dry matter. Water intake per kilogram of dry matter intake (WIDMI) was obtained by dividing total water intake by DMI. Based on these parameters, the amount of water requirement met (AWR) and the amount of water met per kilogram of DMI (AWRDMI) were determined.

### Metabolic parameters

On the first and last days of the experimental period, 30 min before the morning feeding, blood samples were collected from the jugular vein of the lambs using specific tubes for each analysis [Vacutainer^®^]. After collection, the samples were transported to the laboratory, where they were allowed to rest at room temperature for approximately 20 min and then centrifuged for 10 min (centrifuge model 80-2B-15ML, Centribio) at 4,000 rpm (1,800 × g) (Ramalho et al. 2012). Following this procedure, plasma and serum were transferred to properly labeled 1.5 mL microtubes and stored in a freezer at -20 °C for laboratory analyses.

The analyses were performed using an automatic hematology analyzer (model Hematoclin 3.7 - R19114 - Bioclin^®^) and an automatic biochemical analyzer (model Bioclin 6000 - R19179 - Bioclin^®^). The blood parameters analyzed were: red blood cells, hemoglobin, hematocrit, leukocytes, segmented cells, eosinophils, typical lymphocytes, platelets, creatinine, glucose, aspartate aminotransferase (AST), alanine aminotransferase (ALT), and urea.

Spot urine samples were collected from the animals on the last day of the experimental period, four hours after the first feeding, during spontaneous urination or induced by temporary nasal occlusion for 10 to 20 s (Garcia-Navarro 2005). Subsequently, the urine was filtered, and 10 mL aliquots were taken and stored in labeled plastic containers for urinalysis, in order to prevent cellular degradation and bacterial proliferation.

The analyses were performed using physicochemical and microscopic methods, following the laboratory’s standard procedures. The estimated daily urine volume from the spot samples was calculated based on the average creatinine excretion rate obtained for each animal. The physical examination of urine included the evaluation of volume, color, odor, aspect, and density. In the chemical examination, urine pH was measured using a pH meter (Check-Mite^®^), calibrated every five animals with buffer solutions of pH 4.0 and pH 7.0.

The urinary sediment examination was performed after centrifuging the samples and observing them under an optical microscope (Excelsa II^®^, Fanen, São Paulo/SP, Brazil). A 5 mL urine sample was centrifuged in conical tubes at 700 × g for 5 min (Kaneko et al. 2008). The sediment analysis followed the technique described by Valenciano and Cowell (2014). After centrifugation, 0.5 mL of urine was retained for sediment evaluation, which included leukocyte identification.

### Statistical analyses

The obtained data were initially tested for statistical assumptions, including normality and homoscedasticity. After meeting these requirements, an analysis of variance was performed at a 5% significance level using the PROC MIXED procedure (SAS Inst. Inc., Cary, NC, USA).

Physiological parameters were analyzed as repeated measures over time. The most appropriate covariance structure was selected based on the corrected Akaike Information Criterion (AICC) and the Bayesian Information Criterion (BIC). The best model was the one with the lowest AICC or BIC values. The covariance matrix that best fit the dataset was the first-order ante-dependence structure (ANTE(1)) for body temperature and the first-order autoregressive structure (AR(1)) for rectal temperature and respiratory rate.

## Results

Table 2 presents the data related to intake and ingestive behavior of the lambs. It was observed that the supply of DFP resulted in a decrease in DMI and NDFI (*P* ≤ 0.025). Regarding ingestive behavior, except for feeding time (*P* ≥ 0.855), FEDM (*P* = 0.191), and RUMNDF (*P* = 0.202), all other parameters evaluated were affected by the diets (*P* ≤ 0.021). The time spent on rumination and idle was higher and lower, respectively, in animals fed the DTH diet. In terms of efficiencies, lambs fed DFP showed greater RUMDM, whereas those receiving DTH exhibited higher FENDF values.

**Table 2:** Intake and ingestive behavior of lambs fed diets containing faveira pod meal as a replacement for Tifton-85 hay

Item	Diets		SEM	*P* value
	DTH	DFP		
DMI, g/day	1107.2	940.3	42.58	0.025
NDFI, g/day	429.4	220.8	33.88	< 0.001
Feeding time, h/day	3.65	3.7	0.68	0.863
Feeding time, % time	15.2	15.5	0.69	0.855
Rumination time, h/day	6.8	2.8	0.16	< 0.001
Rumination time, % time	28.6	11.9	2.84	< 0.001
Idle time, h/day	13.4	17.4	0.65	< 0.001
Idle time, % time	56.1	72.5	2.71	< 0.001
FEDM, g MS/h	307.5	261.3	18.32	0.191
FENDF, g FDN/h	119.9	61.0	10.19	< 0.001
RUMDM, g MS/h	166.9	396.1	52.11	0.021
RUMNDF, g FDN/h	63.7	89.9	9.68	0.202

DTH, diet with Tifton-85 hay; DFP, diet with faveira pod meal replacing Tifton-85 hay

DMI, dry matter intake; NDFI, neutral detergent fiber intake; FEDM, feeding efficiency in DM intake; FENDF, feeding efficiency in NDF intake; RUMDM, rumination efficiency in DM intake; RUMNDF, rumination efficiency in NDF intake

SEM, standard error of the mean

**Fig. 1:**
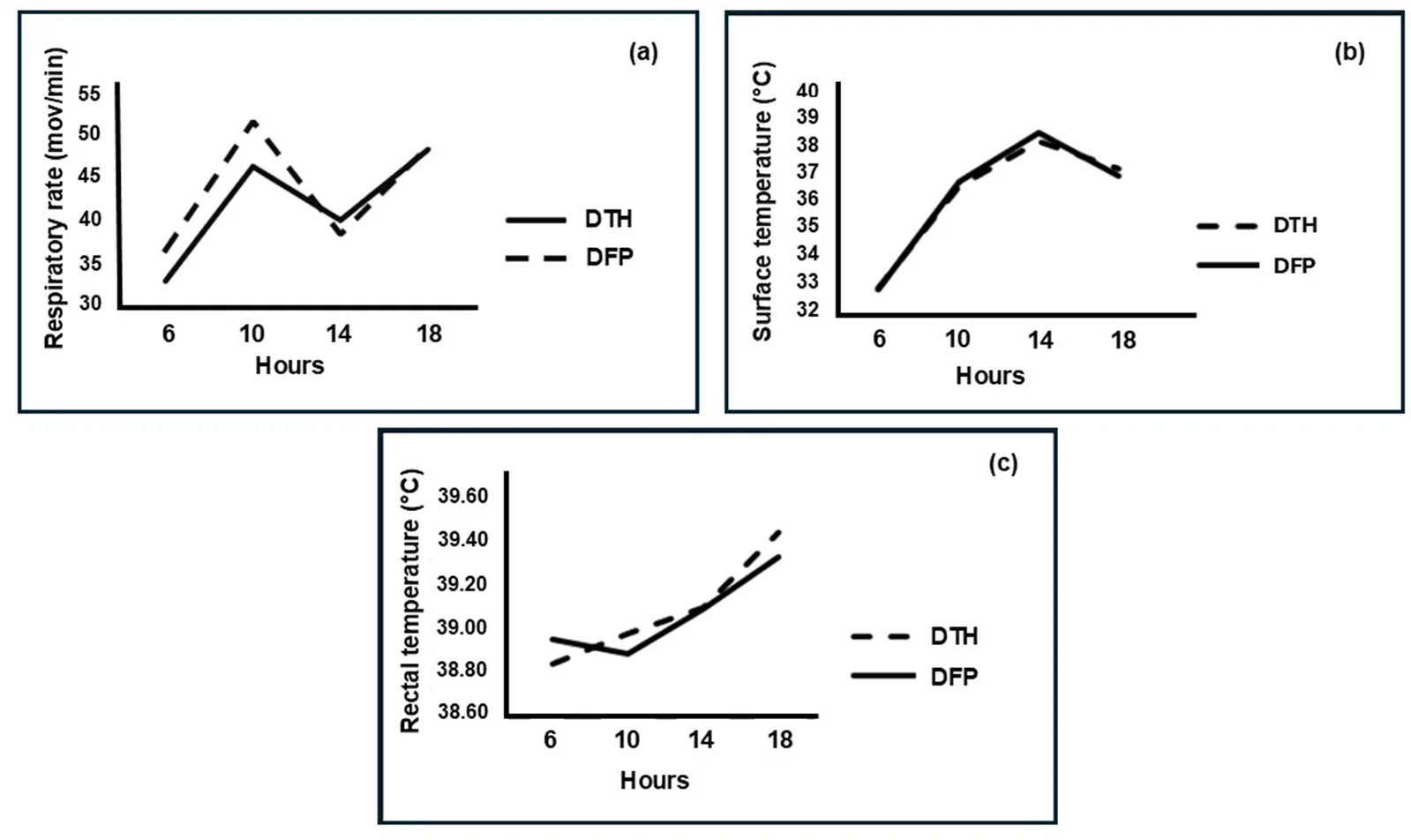
Respiratory rate (**a**), surface temperature (**b**), and rectal temperature (**c**) of lambs fed diets containing faveira pod meal as a replacement for Tifton-85 hay, as a function of sampling time

The physiological parameters of the lambs were not affected by the experimental diets (*P* ≥ 0.209; Table 3). Moreover, no interaction between diet and sampling time was observed for these parameters (*P* ≥ 0.057). However, sampling time influenced all physiological parameters evaluated (*P* < 0.001; Fig. 1). As shown in Table 4, the diets affected WI (*P* = 0.048), WIF (*P* = 0.029), and AWR (*P* = 0.045), with the provision of DFP leading to a decrease in the values of these parameters.

**Table 3:** Physiological parameters of lambs fed diets containing faveira pod meal as a replacement for Tifton-85 hay

Item	Diets		SEM	*P* value		
	DTH	DFP		Diet	Hour	D × H
RR, mov/min	42.84	45.37	1.48	0.872	< 0.001	0.106
ST, °C	36.36	36.54	0.31	0.530	< 0.001	0.355
RT, °C	39.29	39.11	0.04	0.209	< 0.001	0.057

DTH, diet with Tifton-85 hay; DFP, diet with faveira pod meal replacing Tifton-85 hay

RR, respiratory rate; ST, surface temperature; RT, rectal temperature

SEM, standard error of the mean; D × H, effect of the interaction between diet and sampling time

**Table 4:** Water intake of lambs fed diets containing faveira pod meal as a replacement for Tifton-85 hay

Item, kg/d	Diets		SEM	*P* value
	DTH	DFP		
WI	3.65	2.77	0.24	0.048
WIF	0.18	0.15	0.01	0.029
AWR	3.84	2.92	0.25	0.045
WIDMI	3.29	2.94	0.16	0.301
AWRDMI	3.45	3.11	0.16	0.312

DTH, diet with Tifton-85 hay; DFP, diet with faveira pod meal replacing Tifton-85 hay

WI, water intake; WIF, water intake from feed; AWR, amount of water requirement met; WIDMI, water intake per kilogram of dry matter intake, AWRDMI, amount of water met per kilogram of dry matter intake

SEM, standard error of the mean

Among the evaluated blood parameters (Table 5), the diets affected only glucose (*P* = 0.005) and urea (*P* = 0.023) concentrations. The provision of DFP resulted in higher glucose concentrations, whereas the use of DTH led to an increase in urea concentration. Regarding the urinary parameters presented in Table 6, it was observed that lambs fed the DFP diet showed lower urine pH values (*P* = 0.004). The remaining parameters were not affected by the diets (*P* ≥ 0.508), except for urine color, which was golden-yellow in animals fed the DFP diet and light-yellow in those receiving the DTH diet.

**Table 5:** Blood parameters of lambs fed diets containing faveira pod meal as a replacement for Tifton-85 hay

Item	Diets		SEM	*P* value
	DTH	DFP		
Red blood cells (millions/mm^3^)	9.61	9.03	0.38	0.449
Hemoglobin (millions/mm^3^)	9.63	9.05	0.38	0.453
Hematocrit (millions/mm^3^)	28.75	26.92	1.11	0.423
Leucocytes (millions/mm^3^)	8.18	7.73	0.40	0.588
Segmented cells (millions/mm^3^)	39.92	40.33	1.67	0.907
Eosinophils (millions/mm^3^)	3.08	5.08	0.61	0.094
Lymphocytes (millions/mm^3^)	56.58	53.42	1.86	0.417
Platelets (millions/mm^3^)	288.60	271.07	13.91	0.538
Creatinine (mg/dL)	0.59	0.62	0.02	0.411
Glucose (mg/dL)	68.08	77.42	2.22	0.005
AST (U/L)	139.92	120.17	6.21	0.109
ALT (U/L)	38.00	36.42	5.00	0.880
Urea (mg/dL)	44.33	35.25	2.07	0.023

DTH, diet with Tifton-85 hay; DFP, diet with faveira pod meal replacing Tifton-85 hay

AST, aspartate aminotransferase; ALT, alanine aminotransferase

SEM, standard error of the mean

**Table 6:** Urinalysis of lambs fed diets containing faveira pod meal as a replacement for Tifton-85 hay

Item	Diets		SEM	*P* value
	DTH	DFP		
Volume (ml)	4.68	4.51	0.13	0.508
Color	Light-yellow	Golden-yellow	-	-
Odor	Normal	Normal	-	-
Aspect	Clear	Clear	-	-
Density (mg/dl)	1.02	1.03	0.42	0.769
pH	7.46	6.33	0.22	0.004
Leucocytes (millions/mm^3^)	9.83	9.27	1.07	0.807
Crystals	Absent	Absent	-	-

DTH, diet with Tifton-85 hay; DFP, diet with faveira pod meal replacing Tifton-85 hay

SEM, standard error of the mean

## Discussion

The lower NDFI observed with the provision of DFP is due to the lower concentration of this component in the diet, which results from the replacement of Tifton-85 hay with faveira pod meal. According to Lima et al. (2024), faveira pods contain approximately 4.5 times less NDF than Tifton-85 hay, which explains the reduction in NDF levels observed in the DFP diet. It is noteworthy that the decrease in NDF content in DFP resulted in a higher NFC concentration in this diet. This outcome is highly relevant since the greater NFC concentration increases the animal’s energy supply due to its high digestibility (Van Soest 1994; Villalba et al. 2021). In the literature, faveira pod meal is recognized for its richness in NFC, and its inclusion in ruminant diets has been shown to enhance the digestibility of this fraction (Araújo et al. 2019; Gomes et al. 2024).

Lambs fed the DFP diet showed higher blood glucose concentrations, which can be attributed to the greater energy supply provided by the NFC in this diet. Araújo et al. (2019) reported a trend toward increased plasma glucose values with the rising inclusion of faveira pod meal in the diet of dairy goats. In the present study, the higher glucose availability via NFC in the DFP diet resulted in greater energy density, which likely contributed to the reduction in DMI, suggesting that the animals needed to ingest less feed to meet their energy requirements. In a study with dairy cows, Wei et al. (2018) also reported a decrease in DMI as dietary NFC levels increased.

The higher plasma urea concentration observed with the use of the DTH diet suggests greater ruminal ammonia escape, possibly due to the lower NFC concentration in this diet. In contrast, animals fed the DFP diet showed lower plasma urea concentrations, which may be related to the higher NFC content. According to Hanlon et al. (2023), proper synchronization between protein and carbohydrates is essential to optimize nutrient utilization, promoting greater microbial protein synthesis and, consequently, increased animal productivity. Thus, the higher NFC supply in the DFP diet favored dietary protein utilization, enhancing nitrogen retention and reducing nitrogen excretion into the environment.

Although lambs fed the DFP diet showed lower DMI, feeding time did not differ between groups. It was expected that animals fed the DTH diet would spend more time eating, since this diet had a lower energy concentration and contained larger particles from Tifton-85 hay, requiring more chewing time during feeding. However, these animals compensated for the similar feeding time by increasing rumination time, a typical behavior when good-quality fiber is available. In addition, it is likely that providing the DFP diet led the animals to spend more time selecting the feed due to the presence of whole corn grain, resulting in a feeding time similar to that observed in the DTH group. This behavior also explains the lower FEDM (numerically) and FENDF (statistically), since the DMI and NDFI values were lower in this treatment.

The longer rumination time observed in the DTH diet is justified by its higher forage NDF content, which positively influences rumination time (Beauchemin 2018; Grant and Cotanch 2023). Furthermore, according to the *Penn State* particle separator, approximately 63% of the hay particles were larger than 8 mm, requiring additional mastication and consequently increasing rumination time (Zebeli et al. 2007).

It is important to highlight that the additional mastication during rumination plays a key role in reducing feed particle size, allowing for microbial colonization and ruminal digestion, which are essential to meet nutritional requirements (Beauchemin 2018). Due to the shorter rumination time observed, the use of DFP resulted in higher RUMDM (statistically) and RUMNDF (numerically) values, indicating that lambs fed this diet were more efficient, as they ruminated a greater amount of DM and NDF in less time. Similar results were reported by Batista et al. (2020), who observed that including 33.3 and 67% of faveira pod meal in the diet of lactating goats also increased RUMDM and RUMNDF values.

The positive effects of DTH on WI, WIF, and AWR values can be partially explained by the higher feed intake promoted by this diet, which, in addition to increasing the water supply through feed moisture, also required greater WI to support intermediate metabolism. In the literature, several studies have suggested a positive correlation between DMI and WI (Holter and Urban 1992; Dado and Allen 1994; Lukas et al. 2008). Although WI was lower in DFP, AWRDMI did not differ, suggesting that lambs fed this diet consumed the necessary amount of water relative to their DMI.

It is important to highlight that the average WI observed in this study (3.32 kg/d) was higher than the value recommended by the NRC (2007) for sheep. This difference is attributed to the climatic conditions of the study region, characterized by high temperatures that increase WI due to greater evaporative water losses. These losses are also associated with the lower water content of high-concentrate diets (Loiola Filho et al. 2012).

In both treatments, urinary density values remained within the normal range for sheep (1.015 to 1.045), as described by Antonelli et al. (2012). Regarding pH, it is worth noting that the values obtained were also within the range recommended for this species (Hendrix 2005). This result is highly relevant, indicating that the dietary fiber fulfilled its nutritional role without predisposing the animals to acidosis. Diets rich in concentrates, such as those used in the present study, tend to decrease urinary pH (Araújo et al. 2009). In addition, the lower urinary pH observed in lambs fed the DFP diet may also be associated with the lower WI promoted by this treatment, since, according to Navarre (2007), reduced WI can lower urinary pH.

The faveira pod meal can replace Tifton-85 hay in high-concentrate diets for finishing lambs, as it not only does not impair ingestive behavior and physiological parameters but also improves the utilization of dietary nutrients.

## Supplementary Information

Below is the link to the electronic supplementary material.


Supplementary Material 1


## Data Availability

The data that support this paper are available from the corresponding author upon request.
