# Supplementary material for: Parkia platycephala pod meal as a total replacement for Tifton-85 hay in high-concentrate diets for lambs: ingestive behavior, physiological, and metabolic parameters
Source: Trop Anim Health Prod. 2026 Jan 30;58(2):75. doi: 10.1007/s11250-026-04850-z (PMC12858488; doi:10.1007/s11250-026-04850-z)
Supplement: Supplementary file 1 — Supplementary Material 1 [file 11250_2026_4850_MOESM1_ESM.docx]

Chemical composition of the experimental ingredients

| Ingredients (%) | Tifton-85 hay | Faveira pod meal | Corn | Soybean meal | Wheat bran |
| --- | --- | --- | --- | --- | --- |
| Dry matter | 88.75 | 87.58 | 88.75 | 86.45 | 88.01 |
| Ash | 7.17 | 2.62 | 2.41 | 7.47 | 5.32 |
| Neutral detergent fiber | 75.41 | 14.54 | 11.62 | 16.16 | 33.02 |
| Crude protein | 8.20 | 9.20 | 9.87 | 43.83 | 14.14 |
| Ether extract | 2.79 | 4.63 | 8.57 | 6.19 | 4.70 |
| Total carbohydrates | 81.83 | 83.54 | 79.15 | 42.51 | 75.84 |
| Non-fibrous carbohydrates | 6.42 | 69.01 | 67.53 | 26.35 | 42.82 |

Physical characterization of the experimental diets

| Ingredients | *Penn State* fractions | | | |
| --- | --- | --- | --- | --- |
|  | >19mm | 9 a 19mm | 4 a 8mm | <4mm |
| Tifton-85 hay | 31.52 | 31.52 | 4.35 | 33.70 |
| Ground corn | * | * | 47.79 | 51.88 |
| Soybean meal | * | * | 5.11 | 94.47 |
| Wheat bran | * | * | 9.07 | 90.08 |
| Faveira pod meal | * | * | 9.80 | 89.80 |
| Whole corn grain | * | 94.10 | 5.28 | 0.31 |
| DTH | * | 0.22 | 24.94 | 70.24 |
| DFP | * | 22.27 | 6.48 | 70.24 |

DTH, diet with Tifton-85 hay; DFP, diet with faveira pod meal replacing Tifton-85 hay
